# Repair of Perineal Hernia Following Abdominoperineal Excision with Biological Mesh: A Systematic Review

**DOI:** 10.3389/fsurg.2016.00049

**Published:** 2016-09-05

**Authors:** Sunil K. Narang, Nasra N. Alam, Ferdinand Köckerling, Ian R. Daniels, Neil J. Smart

**Affiliations:** ^1^Exeter Surgical Health Services Research Unit (HeSRU), Royal Devon and Exeter Hospital, Exeter, Devon, UK; ^2^Department of Surgery, Center for Minimally Invasive Surgery, Vivantes Hospital, Academic Teaching Hospital of Charité Medical School, Berlin, Germany

**Keywords:** perineal hernia, abdominoperineal excision, biologic mesh, biosynthetic mesh

## Abstract

**Introduction:**

Perineal hernia (PerH) following abdominoperineal excision (APE) procedure is a recognized complication. PerH was considered an infrequent complication of APE procedure; however, PerH rates of up to 45% have been reported in recent publications following a laparoscopic APE procedure. Various methods of repair of PerH with the use of synthetic meshes or myocutaneous flap have been described, although there is no general agreement on an optimal strategy. The use of biological meshes for different operations is growing in popularity, and these have been promoted as being superior and safer when compared to synthetic meshes. Although the use of biologics is becoming popular claims of better outcomes are largely unsupported by evidence. The aim of this systematic review is to evaluate the currently available evidence supporting the use of biologic or biosynthetic meshes for the repair of PerH that develop following an APE.

**Methods:**

A systematic review of all English language literature relevant to repair of PerH following APE with biologic or biosynthetic mesh published between January 1, 2000 and July 31, 2016 was carried out using MEDLINE, EMBASE, and the Cochrane Library of Systematic Reviews for relevant literature. Searches were performed using a combination of Medical Subject Headings (MeSH) terms and text words “PerH,” “APE,” “morbidity,” “biologics,” “biosynthetic,” and “hernia.” Studies in which the use of biological meshes was not reported were excluded from the review. Various outcome measures, including operative technique, complication rates, recurrence rates, type of mesh, management of recurrences, and risk factors, were extracted. Oxford Centre for Evidence-based Medicine – Levels of Evidence (March 2009) was used to assess the quality of evidence.

**Results:**

The systematic review of the literature identified three case reports, four case series, and one pooled analysis that were included in the final review. Overall, these studies were of poor quality providing level 4 evidence. Various different approaches and techniques of repair of PerH were described; however, it was difficult to extract information with regard to the primary and secondary outcome measures.

**Conclusion:**

There is no general agreement to the optimal operative strategy to repair PerH following an APE. There is insufficient evidence to recommend any specific operative approach or repair technique for PerH following an APE.

## Introduction

The finding of a perineal hernia (PerH) following an abdominoperineal excision (APE) is a recognized complication; however, it is unclear as to how frequently this occurs. Until a few years ago, they were considered to be an infrequent complication following an APE and prevalence rates of 0.6–7% were reported (1–6). However, the surgical management of rectal cancer has evolved over the recent years with the acceptance of the principle of total mesorectal excision (TME) and the recognition of the importance of a clear surgical resection and avoidance of tumor perforation during an APE (7). This procedure has evolved into the extra levator abdominoperineal excision (eLAPE) with the potential surgical resection margin information being identified through MRI staging (7, 8). This has resulted in a reduction in circumferential resection margin involvement and intra-operative perforation of the tumor (9). However, an eLAPE creates a wider defect in the pelvic floor leaving only the ischioanal fat and skin for closure of the defect as the entire pelvic floor muscle complex has been excised surrounding the distal rectum. Perineal herniation in this group of patients is increasingly recognized and a recent publication has reported an overall PerH rate of 26% and this can be as high as 45% in those having a laparoscopic eLAPE procedure (10).

A PerH repair may be necessary as the hernia is not only painful but can also result in urinary dysfunction or bowel obstruction causing impairment of daily activities of living. Various methods or repair have been described in the literature, including primary tissue repair, synthetic mesh, biological mesh, and myocutaneous flaps. This repair can be facilitated by either using an abdominal and/or perineal approach although none of the described repairs are well established.

The use of synthetic meshes is associated with problems, such as mesh infection, chronic inflammation, and foreign body reaction. If bowel is in direct contact with the synthetic mesh used for a PerH repair, then there is a risk of adhesions and erosion into the bowel wall by the mesh. Biologic and biosynthetic meshes were developed to overcome such problems. The role of biologic mesh for primary reconstruction of the pelvic floor after eLAPE has been the subject of a systematic review, and this was considered a promising technique for improving wound healing and complication rates comparable to other techniques (11). Biologic meshes have been used recently as an alternative for repairing PerH following an eLAPE. The biologic mesh acts not only as a structural support for the hernia repair but also as a scaffold allowing the ingrowth of native fibroblasts, which in turn lay down the fibrous tissue and promote tissue remodeling (12). The aim of this systematic review is to evaluate the currently available evidence supporting the use of biologic or biosynthetic meshes for the repair of PerH.

## Methods

### Search Strategy

A systematic review of all English language literature relevant to the repair of a PerH following an APE with biologic or biosynthetic mesh published between January 1, 2000 and July 31, 2016 was carried out using MEDLINE (PubMed and Ovid), EMBASE (Ovid), and the Cochrane Library of Systematic Reviews/Controlled Trials for relevant literature. Searches were performed using a combination of Medical Subject Headings (MeSH) terms and text words “*perineal hernia*,” “abdominoperineal excision,” “*morbidity*,” “*biologics*,” “*biosynthetic*,” and “*hernia*.” All randomized/non-randomized, controlled/non-controlled clinical trials, prospective observational studies, clinical registry data, retrospective case series, and case reports that reported on repair of PerH following APE were included for analysis. Conference abstracts, letters, technical notes, and commentaries were excluded. In addition, bibliographies from the papers requested were manually checked to identify additional relevant papers.

### Study Selection

Titles and abstracts of the identified studies were screened by the main reviewer Sunil K. Narang and independently checked by Nasra N. Alam. Studies that were irrelevant were rejected. The full texts of identified papers were independently assessed by two reviewers (Sunil K. Narang and Nasra N. Alam) to determine whether they met the predetermined inclusion/exclusion criteria. Disagreements were resolved by discussion or adjudication by the senior author (Neil J. Smart).

### Inclusion Criteria

All studies should have been published in print or electronic format between 1 January, 2000 and July 31, 2016. Only adult patients undergoing PerH repair following APE were included in the review. An APE may have been performed as an open procedure, laparoscopic, hand-assisted or robot-assisted. PerH repair may have been done using open, laparoscopic, or combined approach. The diagnosis of PerH may have been established based on clinical examination or cross-sectional imaging.

### Exclusion Criteria

Studies on the pediatric population or using synthetic mesh or myocutaneous flaps were excluded from this review. Diagnosis of PerH established on the basis of patient-reported symptoms of PerH or telephone or postal follow-up were excluded from the review.

### Outcomes

The primary outcome measure of the systematic review was to assess the recurrence of PerH following repair with biologic or biosynthetic meshes. Other factors, such as time interval to development of PerH and diagnostic definition of PerH (clinical, cross-sectional imaging, patient-reported or telephone interview), were also noted.

The secondary outcome measures recorded were:

1. Complications following repair.
2. Management of recurrences.
3. Patient-reported outcome measures or quality of life scores.

### Definitions

Clinically, a PerH is defined as a palpable bulge in the perineum associated with protrusion of intra-abdominal or pelvic viscera through the defect in the pelvic floor fascia and musculature. Radiographic definition of PerH unclear as the landmarks for defining the pelvic floor are not universally agreed.

### Quality Assessment

Oxford Centre for Evidence-based Medicine – Levels of Evidence (March 2009) was used to assess the quality of evidence (13).

### Data Extraction (Selection and Coding)

Data on the study type, number of patients treated, length of follow-up, cross-sectional imaging, and symptoms from PerH were extracted from the included studies by the reviewers. These data were extracted separately by reviewers (Sunil K. Narang and Nasra N. Alam) to guard against reviewer bias. Any discrepancies were resolved by adjudication by the senior author Neil J. Smart. All data and results of statistical tests were extracted from the papers and entered into an electronic data sheet (Microsoft Excel). For particular outcomes that were to be evaluated, if the data were not specifically reported, they were regarded as not reported or missing and no assumptions were made regarding the missing data.

### Statistical Analysis

There was a significant heterogeneity in the included studies in the study design, intervention design, study cohorts, and outcome measures. A weighted analysis of variables for risk factors for PerH development was not possible because of the lack of both uniformity and the quantity of the data reported. For this reason, a meta-analysis of the data could not be performed; therefore, primary and secondary outcome measure parameters are expressed as a range.

## Results

**Figure 1:**
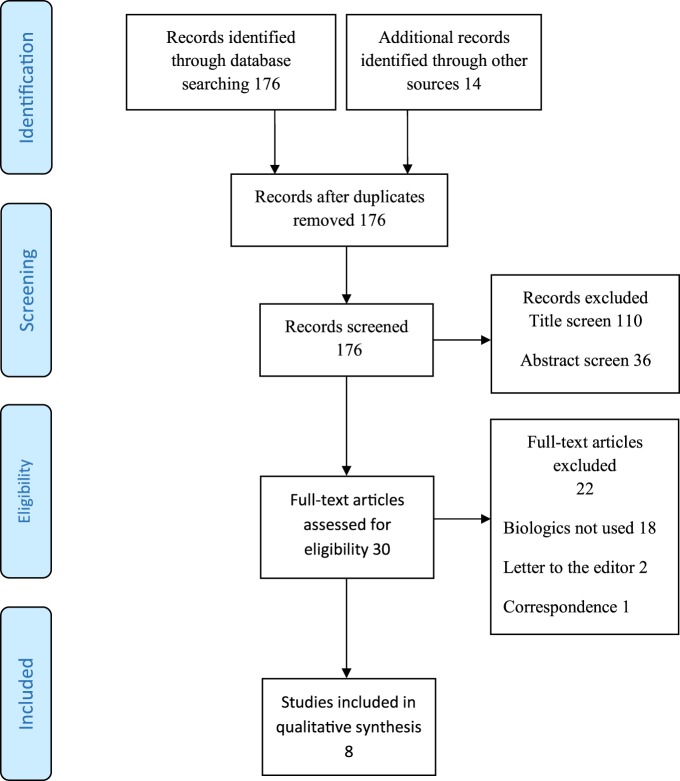
**PRISMA flow diagram outlining study selection**.

A total of 190 potential articles were identified from the initial literature search. After removal of duplicate articles 176 articles remained (Figure 1). Using the inclusion criteria described above, 146 articles were eliminated on title and abstract review. Full text articles were obtained for 30 articles out of which 22 articles were rejected, as they did not meet the inclusion criteria. Eight articles were included for final analysis. Out of the eight articles that were included, three were case reports, four were case series, and one article was a pooled analysis. The quality assessment of the included studies is presented in Tables 1–3. The level of evidence based on the Oxford Centre for Evidence-based Medicine (March 2009) was 4 at best. The pooled analysis included all publications from 1944 to 2010 and has probably included data from Skipworth et al. (14) and de Campos et al. (15). There was a significant variation among studies in their description of diagnostic method, selection criteria, operative technique, type of mesh used, and duration of follow-up, recurrence rates, complications, and management of recurrences. None of the studies in the review used the Clavien Dindo grading of post-operative complications (16).

**Table 1:** **Case reports**.

Reference	*n*	Study period	M:F	Age (years)	Treatment of primary disease	Approach to perineal hernia repair	Mesh type	Follow-up	Outcome	Complications
Ong and Miller (17)	1	12 months after APR		72	Neoadjuvant CRT	Transperineal using Mitek suture anchors	Acellular porcine dermal mesh (Permacol)	6 months	No recurrence	Nil
Kathju et al. (18)	1	2011	M	56	Neoadjuvant CRT, APR	Abdominoperineal Mesh anchored anteriorly to pubic bone using Mitek suture anchors	Human-derived acellular dermal graft (Derma Matrix)	1 year	No complications or recurrence	NR
Skipworth et al. (14)	1	2006	M	46	Pre op CRT	Perineal approach Trendelenburg lithotomy positon	Porcine collagen matrix	18 months	No recurrence	NR

**Table 2:** **Case series**.

Reference	*n*	Study period	M:F	Age	Treatment of primary disease	Approach to perineal hernia repair	Mesh type	Follow-up	Outcome	Complications
Musters et al. (19)	15	50 months	9:6	62 ± 11 years mean	Conventional APR (*n*, %) 5 (33) Extralevator APR (*n*, %) 5 (33) Ischio-anal APR (*n*, %) 4 (27) Intersphincteric APR (*n*, %) 1 (7)	Transperineal 14 Laparoscopic Omental plasty + Transperinal 1	Permacol™ 3 Strattice™ 12 Myocutaneous flap + Biological mesh 3	17 months median (IQR 12–24)	Clinical recurrence 7 (47%)	Wound infection 3 patients

Sayers et al. (10)	14/54	54 months	40:14	69.5 years median (31–90)	eLAPE 20 Neo-CRT 52 Biological mesh 2 Myocutaneous flap 6 (5 rectus and 1 gracilis) Simple suture in 46	Not reported	Biologic mesh 5/8 Myocutaneous flap 3/8	57.5 months, median (29–61)	Biologic mesh, 1/5 had recurrence Myocutaneous flap, 1/3 had recurrence	NR

Abbas and Garner (20)	7	Over 66 months	4:3	64 years median (44–77)	0.5 after lap APER 1 had gluteal rotation flap All had RT 1 had adjuvant CT	Lap repair 5 Lap converted to open 1 Perineal approach 1 (sublay)	Synthetic composite 4 Biological 2 Direct suture repair 1	25 months (16–64)	No recurrences	NR

de Campos et al. (15)	7	1995–2004	NR	NR	35 patients in one center had pre op CT. 4/35 developed PERH 3 patients from another center		Dura mater patch via laparotomy 1 Bovine pericardium 1 via perineal approach Bovine pericardium via abdominal approach 1 Conservative 1	NR	NR	NR

**Table 3:** **Pooled analysis evidence**.

Reference	*n*	Study period	M:F	Age	Treatment of primary disease	Approach to perineal hernia repair	Mesh type	Follow-up	Outcome	Complications
Mjoli et al. (21)	43	1944–2010	23:20	63 years mean (10 SD, range 45–89)	RT 18 Open APE24 (55.8%) Open APE + coccyx 4 (9.3%)	Perineal 22 Open abdominal in 11 Open abdominoperineal 3	**Perineal** Non-absorbable 3 Composite 1	NR	Primary recurrence 13 Second recurrence 3	Perineal wound breakdown 12%
					Laparoscopic APE 9 (20.9%) Laparoscopic APE + posterior vaginal wall 3 (7.0%)	Laparoscopic 5 Laparoscopic-perineal 2	Biological 4 Non-specific 1		Recurrence rate: 5/25 synthetic or biological mesh	
					Staged Lahey procedure 1 (2.3%) Staged Lahey procedure + coccyx 1 (2.3%) Open APE + perineal colostomy 1 (2.3%)		**Open abdominal** Absorbable mesh 1 Non-absorbable 3 Biologic 3		6/12 primary closure; 2/6 remaining techniques Recurrences repaired: synthetic or biologic mesh 6	
							**Open Abd-Perineal** Non-absorbable 3		Primary closure 5 Gluteal/Gracilis flap 4	
							**Laparoscopic** Composite 5			
							**Laparoscopic-perineal** Non-absorbable 1 Composite 1			

In the three case reports, there were no recurrences following repair of PerH on follow-up ranging from 12 to 18 months (11, 13, 14). Of the four case series, the duration of follow-up and final outcome was not reported in one publication (4, 8, 12). In the remaining three case series, different types of cross and non-cross-linked biologic meshes were used and some patients underwent myocutaneous flap repair and/or omentoplasy in addition to the mesh repair.

The operative technique to repair a PerH varied significantly ranging from perineal repair, open abdominal repair, or combined approach with or without the use of laparoscope. The type of biological mesh used was not reported in three studies. Others described the use of additionally cross-linked acellular porcine collagen (Permacol™), non-cross-linked porcine collagen (Strattice™), Human-derived Acellular Dermal graft (DermaMatrix), Dura mater patch, and Bovine pericardium. Complications following repair were not reported in any of the case reports although the pooled analysis reported a perineal wound breakdown rate of 12% with the use of all different types of synthetic and biologic meshes. Management of recurrence of PerH was reported by Musters et al. and four recurrent PerH were repaired using prolene mesh (19). None of the studies used patient-reported outcome measures to assess the impact of surgery on the quality of life of the patients.

## Discussion

A PerH is an incisional hernia through the pelvic floor, which results in protrusion of abdominal or pelvic viscera. There is no universally accepted clinical or radiological definition of a PerH. They are typically diagnosed based on symptoms, such as an expansile cough impulse in the perineum, which is not only uncomfortable but can also cause bowel or bladder symptoms. In the majority of cases, this hernia remains asymptomatic and may be incidentally detected on cross-sectional imaging performed for oncologic follow-up. It is vital that there should be a universally agreed clinical and radiological definition of PerH in order to make meaningful comparison between studies.

Most publications reporting PerH repair following APE are either individual case reports or small retrospective case series with relatively short follow-up. This review includes mostly small case series over a long period of time reporting different techniques performed by different surgeons. These papers are focused on the description of successful technique and are, therefore, prone to publication bias. It appears that myocutaneous flap techniques have a role in repair of PerH when the operative field has been severely damaged by irradiation and can provide a well-vascularized tissue for repair (19, 22). None of the studies compared the use of biologics with either prosthetic mesh or myocutaneous flaps and, therefore, findings of individual studies are difficult to interpret.

Some of the risk factors that may predispose an individual to the development of PerH include female gender, previous hysterectomy, coccygectomy, pre-operative pelvic irradiation, post-operative wound infection, a long small bowel mesentery, high BMI, smoking, and non-closure of the pelvic peritoneum (1, 6, 21, 23, 24). Patients with rectal cancer may have been treated with neoadjuvant chemoradiotherapy. The role of these risk factors has not been evaluated in these studies.

The decision to repair PerH is based on the symptoms, fitness of the patient and oncological stage. Repair may be performed via abdominal, perineal, or combined approach. Laparoscopy has been used in patients with reasonable access. There is no evidence to support the use of any particular approach. The pooled analysis by Mjoli et al. reported 22 perineal repairs, 11 open abdominal operations, 3 combined abdominoperineal approach, 5 laparoscopic repairs, and 2 laparoscopic-perineal procedures (21).

Within the last decade, the eLAPE procedure has become increasing popular and has led to reduction in the circumferential margin positivity rate. However, due to the wider resection of the pelvic floor, the risk of herniation may be higher. The pelvic floor may or may not have been reconstructed using flaps/meshes, etc. There is evidence that laparoscopy results in fewer adhesion in the abdomen and this may contribute to increasing PerH rates as the small and large bowel are free to descend into the pelvis (25, 26). It is likely that the incidence and prevalence of PerH will increase unless there is a much better technique of primary reconstruction of the pelvic floor at the time of APE.

The advent of synthetic absorbable meshes has generated considerable interest within the surgical community. These materials promote fibroblast activity and generate a foreign body reaction. Following complete absorption within 30–90 days, the synthetic material is replaced by collagen rich connective tissue. In this review, only one patient was identified to have undergone repair with an absorbable mesh and this hernia recurred within 16 months (21). The use of biologic mesh for repairing PerH appears attractive as the acellular collagen matrix is believed to allow migration of fibroblasts, neovascularization, and incorporation within the native tissues. This is thought to reduce the risk of wound infection. A recently published case series of 15 patients undergoing PerH repair with porcine acellular dermal mesh reported recurrence rates of 47% after a median follow-up of 17 months (IQR 12–24) (19). However, the low volume, quality of available data, and lack of any comparative studies make it difficult to evaluate the use of biologic meshes as a technique.

## Conclusion

There is no general agreement to the optimal operative strategy to repair PerH following an APE. There is insufficient evidence to recommend any specific operative approach or repair technique for PerH following APE.

## Author Notes

Previous presentations: The data in this paper have been presented at the Consensus Conference on the clinical use of biologic and biosynthetic meshes in abdominal surgery on Wednesday, January 27, 2016 at Hotel Ellington, Nürnberger Straße 50-55, D-10789 Berlin.

## Author Contributions

SN, FK, ID, and NS designed the search; SN and NA performed the search; FK, ID, and NS contributed analytic tools; SN and NA analyzed the data. SN wrote the first draft paper. All authors contributed to revision of the manuscript.

## Conflict of Interest Statement

The authors declare that the research was conducted in the absence of any commercial or financial relationships that could be construed as a potential conflict of interest.

## Appendix

### BioMesh Study Group

Ferdinand Köckerling (Chairman), Stavros Antoniou, René Fortelny, Frank A. Granderath, Markus Heiss, Franz Mayer, Marc Miserez, Agneta Montgomery, Salvador Morales-Conde, Filip Muysoms, Alexander Petter-Puchner, Rudolph Pointner, Neil Smart, Marciej Smietanski, Bernd Stechemesser.

### Aim

The BioMesh Study Group has set itself the task of identifying how best to use biological meshes for the various indications. The first step toward achieving that goal is to compile systematic reviews of the different indications on the basis of the existing literature. The available literature sources will be evaluated in accordance with the Oxford Centre for Evidence-based Medicine-Levels of Evidence (March 2009). Next, based on the review findings, corresponding Statements and Recommendations are to be formulated in a Consensus Conference for the use of biological meshes for the different indications. The findings of this study were presented at the Consensus Conference in Berlin in January 2016 as a part of the project undertaken by the BioMesh Study Group. The findings of the Consensus Conference are currently being summarized for a joint publication.
